# Predominance of Multidrug Resistant *Escherichia coli* of Environmental Phylotype in Different Environments of Dhaka, Bangladesh

**DOI:** 10.3390/tropicalmed8040226

**Published:** 2023-04-17

**Authors:** Anindita Bhowmik, SM Tanjil Shah, Sharmistha Goswami, Ahmad Salman Sirajee, Sunjukta Ahsan

**Affiliations:** Department of Microbiology, University of Dhaka, Dhaka 1000, Bangladesh

**Keywords:** *Escherichia coli*, commensal, environmental, phylogenetic groups, multidrug-resistant

## Abstract

Considering the ecological diversity of *E. coli*, the main aim of this study was to determine the prevalence, phylogroup diversity, and antimicrobial susceptibility of *E. coli* isolated from 383 different clinical and environmental sources. In total, varied prevalence was observed of the 197 confirmed *E. coli* that were isolated (human–100%, animal–67.5%, prawn–49.23%, soil–30.58%, and water–27.88%). Of these isolates, 70 (36%) were multidrug-resistant (MDR). MDR *E. coli* was significantly associated with their sources (χ^2^ = 29.853, *p* = 0.001). Humans (51.67%) and animals (51.85%) carried more MDR *E. coli* than other environments. The *eae* gene indicative of recent fecal contamination was not detected in any isolate, indicating that these *E. coli* isolates could be present in these environments for a long time and became naturalized. Phylogroup B1 (48.22%) was the predominant group, being present in all hosts analyzed and with the commensal *E. coli* group A (26.9%) representing the second predominant group. According to chi-square analysis, phylogroup B1 was significantly associated with *E. coli* from humans (*p* = 0.024), soil (*p* < 0.001) and prawn samples (*p* < 0.001). Human samples were significantly associated with phylogroup B1 (*p* = 0.024), D (*p* < 0.001), and F (*p* = 0.016) of *E. coli* strains, whereas phylogroup A (*p* < 0.001), C (*p* < 0.001), and E (*p* = 0.015) were associated with animal samples. Correspondence analysis results also indicated the association of these phylogroups with their hosts/sources. The findings of this study exhibited a non-random distribution of phylogenetic groups, though the diversity index was highest for human *E. coli* phylogroups.

## 1. Introduction

*Escherichia coli,* a common member of the gastrointestinal flora of warm-blooded animals, is considered one of the best indicators of potentially pathogenic bacteria in the environment [1,2]. Since the intestinal tract and feces of warm-blooded animals and humans are the main reservoir of this bacteria, the presence of pathogenic/non-pathogenic *E. coli* strains in foods, drinks, water, and other environments has been used as an indicator of fecal contamination. *E. coli* could be released into the environment through the deposition of fecal material; this bacterium is widely used as an indicator of fecal contamination of waterways. However, *E. coli* was reported to become “naturalized” to soil, sand, sediments, and algae in tropical [3,4], subtropical [5], and temperate environments [6,7,8,9]. It has also been found that the *eae*, a chromosomal gene that encodes the outer membrane protein named intimin, indicates recent fecal contamination in the environment [10]. Hence, the presence of the *eae* can reflect whether an isolate has been resident in a particular environment for a considerable period or has just been shed through feces.

Eight phylogenetic groups of *E. coli* are recognized, with seven belonging to *E. coli* sensu stricto (A, B1, B2, C, D, E, F) and one corresponding to *Escherichia* clade I [11]. *E. coli* strains of the same phylogenetic group share similar phenotypic and genotypic characteristics, disease-causing ability, and ecological attributes [12]. Phylogroup A represents commensal strains; B1 represents environmental strains [13,14]; and phylogroups B2 and D include pathogenic strains of *E. coli* [15]. The presence of phylogroup E, formerly considered a small set of unassigned strains, is now well-recognized [16]. Phylogroup F forms a sister group of phylogroup B2; phylogroup C has been suggested as a group of strains closely related but distinct from phylogroup B1 [11]. Recently, Clermont and colleagues declared that some strains belong to a group intermediate between the F and B2 phylogroups, designated as phylogroup G [17]. Depending on the presence of different marker genes, Clermont and colleagues developed a Quadruplex PCR method to characterize the phylogroups of *E. coli*. The distribution of phylogroups differs based on their ecological niches and life history, including different properties such as their ability to use different sugar sources, their antibiotic-resistance profiles, and also on their rate of growth [11].

Phylogenetic studies help understand *E. coli* and its host and disease [18]. Different phylogroups of *E. coli* are not distributed randomly and were found to be associated with the source of isolation [11]. Phylogenetic grouping of *E. coli* isolated from different sources would help to understand the distribution of such strains in the environment. Therefore, the present study aimed to determine the occurrence and distribution of different phylogroups of *E. coli* in various environments and investigate their antibiotic resistance profile to reflect the species’ adaptability.

## 2. Materials and Methods

### 2.1. Sample Collection and Processing

A total of 383 samples were collected from different sources, including human, fecal specimens of domestic animals, prawns, soil, and water (Table 1, Supplementary File). Clinical samples were collected from the Medinova Diagnostic Centre, Dhaka, Bangladesh, for human sources. Each sample was collected in sterile phosphate buffer saline (PBS pH 7.2) and processed immediately after collection [19,20]. Pre-enrichment was done in buffered peptone water and incubated for 24 h at 37 °C.

### 2.2. Identification of Escherichia coli

After pre-enrichment, an aliquot of pre-enriched culture was plated on selective media such as Eosin Methylene Blue (EMB) and MacConkey agar to isolate characteristic *E. coli* colonies. Single presumptive *E. coli* colonies were tested for Gram-staining (−ve for *E. coli*), citrate utilization (−ve for *E. coli*), glucose and lactose fermentation (+ve for *E. coli*), motility (+ve for *E. coli*), indole production (+ve for *E. coli*), urea hydrolysis (−ve for *E. coli*) and sulfide production (+ve for *E. coli*) following standard procedures [19,20]. The presence of the two molecular marker genes *uspA* and *uidA* confirmed the identification of *E. coli.* A multiplex PCR targeting *uid*A and *usp*A gene was used for *E. coli* confirmation following protocol, as described earlier [21]. A singleplex reaction was used to detect the *eae* gene following the PCR amplification reactions, as described elsewhere [22].

### 2.3. Antibiotic Susceptibility Pattern Determination

The antibiotic susceptibility pattern of *E. coli* was tested as described by Bauer and Kirby disk diffusion method-based standards and interpretive criteria previously established and developed by the Clinical and Laboratory Standards Institute (CLSI) [23]. Eight commercial antibiotic disks (Oxoid, UK) belonging to seven classes of antibiotics were used in this study: Nitrofurantoin (300 µg), Chloramphenicol (30 μg), Tetracycline (30 µg), Amoxicillin-clavulanic Acid (20 µg), Ceftriaxone (30 µg), Ciprofloxacin (5 μg), Gentamycin (10 μg), and Azithromycin (15 µg). The bacterial lawn was prepared on Muller-Hinton Agar (MHA) plate using a freshly grown culture of each *E. coli* isolate. This plate was dried for three to five minutes before the disks were applied. All antibiotic disks were gently pressed onto the agar and incubated at 37 °C. After overnight incubation, the plates were examined for the zone of inhibition. The zone diameters for individual antimicrobial agents were then translated into susceptible, intermediate, or resistant categories according to the CLSI guidelines [23,24].

### 2.4. Determination of Phylogenetic Group

Phylogenetic group determination was done according to Clermont et al. [Clermont 2012] by quadruplex PCR targeting *arpA*, *chuA*, and *yjaA* genes, and the DNA fragment TspE4.C2. To confirm phylogroups A/C and D/E that included E/Clade 1, C, and E-specific PCR was done targeting the *trpA* and *arpA* genes [16]. In all cases, the amplicons were resolved by agarose gel electrophoresis in 1.5% agarose gel in 1X TAE buffer at 80 V for 1–2 h. The strains were assigned to the phylogenetic groups A (*arpA*+), B1 (*arpA*+, TspE4.C2+), B2 (*chuA*+, *yjaA*+)/(*chuA*+, *yjaA*+, TspE4.C2+)/(*chuA*+, TspE4.C2+), C (*arpA*+, *yjaA*+, *trpA*+), D (*arpA*+, *chuA*+)/*arpA*+, *chuA*+, TspE4.C2+), E{*arpA*+, *chuA*+, *arpA* (301 bp)+}/ *arpA*+, *chuA*+, TspE4.C2+, *arpA*(301 bp)+}, or F(*chuA+*).

### 2.5. Statistical Analysis

The Shannon and Simpson diversity indices were calculated (*p* < 0.05) to determine the diversity of phylogroups within different environments [25,26]. The association of *E. coli* phylogroups with the host were studied using the chi-square test. The results were considered significant at *p* < 0.05. Correspondence analysis (CA) was performed to correlate and compare the distribution of *E. coli* isolates of different phylogroups with their source of isolations using RStudio version 1.2.1335. A two-dimensional graph was used to show the relationship between the isolated phylogroups and their sources.

## 3. Results

### 3.1. Identification of the E. coli Isolates 

**Figure 1:**
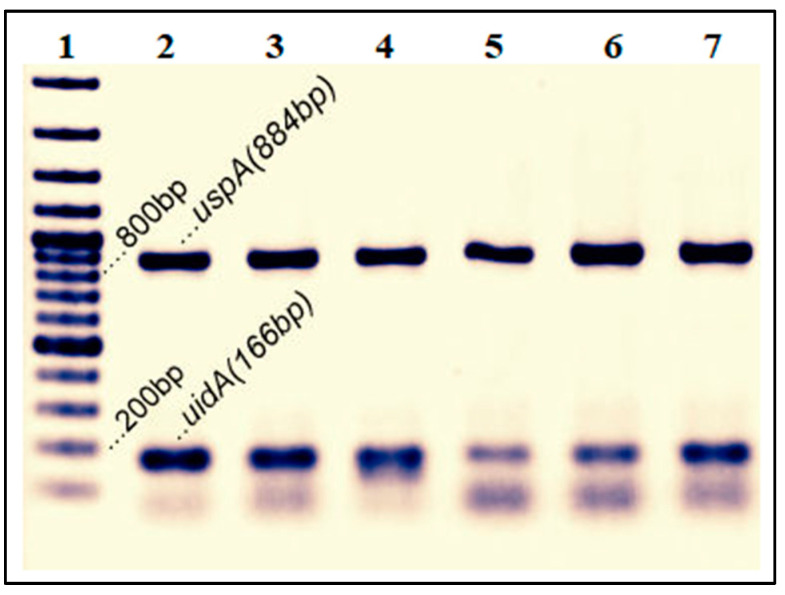
Presence of *uidA* and *uspA* genes in *E. coli* samples. Lane 1, 100 bp Ladder; Lane 2, *Escherichia coli* ATCC 25922 was used as a positive control; lane 3, 4, 5, 6, and 7, isolated experimental isolates.

A total of 197 *E. coli* strains (51.4%) isolated from different sources (n = 383) were presumptively identified through their biochemical characteristics. Presumptively isolated test strains were further confirmed by detecting the presence of *uidA* and *uspA* genes (Figure 1). Prevalence rates of *E. coli* for human, animal, prawns, soil, and water samples were 100.0% (60/60), 67.5% (54/80), 49.23% (32/65), 30.58% (26/85), and 26.88% (25/93), respectively. For non-human samples, the prevalence rate was 42.4%. None of the *E. coli* isolates carried the *eae* gene, which eliminated the likelihood of any recent fecal contamination.

### 3.2. Antimicrobial Resistance Evaluation of E. coli

The phenotypic resistance of the isolated *E. coli* to seven different antibiotics classes is presented in Table 2. The highest resistance was found against tetracyclines (36.0%), followed by ciprofloxacin (33.5%). Moreover, Azithromycin (32.4%) and Amoxicillin-Clavulanic acid (26.9%) resistance phenotypes were dominant among the isolates. Although no single antibiotic was 100% effective for all isolates, 100% sensitivity was found for soil *E. coli* isolates against nitrofurantoin and chloramphenicol.

The multidrug-resistance (MDR) pattern was also analyzed for all 197 isolates (Table 3). In total, 36.0% of the *E. coli* (71/197) showed phenotypic resistance to at least 3 classes of antibiotics tested, and therefore, were considered as MDR *E. coli*. MDR *E. coli* was found to be significantly associated with the source of isolation (χ^2^ = 29.853, *p* = 0.001). Maximum MDR *E. coli* isolates were found in humans (51.67%) and animal sources (51.85%). Twelve and ten *E. coli* isolates showed resistance to at least five different antibiotic classes from human and animal sources, respectively. The lowest MDR *E. coli* was found for soil (7.69%). On the contrary, a moderate number of MDR *E. coli* was present in water (20.0%) and prawn (15.63%).

### 3.3. Phylogroup Diversity of the E. coli Isolates 

In this study, 197 *E. coli* strains isolated from different environmental sources were found to be distributed into 7 phylogenetic groups viz. A, B1, B2, C, D, E, and F, based on the presence of different marker genes that included *arpA*, *chuA*, *yjaA, trpA*, and the DNA fragment TspE4.C2 (Table 4). The most prevalent phylogroup was B1 (48.22%, 95/197), representing *E. coli* of environmental origin. Of the remaining, 26.9% of isolates (53/197) belonged to phylogroup A. Only 2.03% of isolates belonged to phylogroup B2, which generally includes pathogenic *E. coli*, and 8.63% of isolates fell under phylogroup C representing isolates closely related to environmental isolates in terms of the phylogeny. Of these, 9.14% belonged to phylogroup D, and 3.05% of isolates belonged to phylogroup E, as re-confirmed with E-specific PCR. A total of 2.03% of isolates belonged to phylogroup F. Multidrug-resistance profiling of different phylogenetic groups indicated that all phylogroup F isolates were MDR (100%). Additionally, higher percentages of MDR *E. coli* were found for phylogroup C (64.7%, 11/17), D (61.1%, 11/18), and E (66.7%, 4/6).

Table 5 shows the distribution of different phylogroups among the isolates analyzed. Phylogroups B1 and A were present among all the environments investigated, whereas strains from group F were found only in humans.

Phylogroups A (*p* < 0.001), C (*p* < 0.001), and E (*p* = 0.015) were found to be associated with *E. coli* isolated from the animal. On the other hand, phylogroups B1 (*p* = 0.024), D (*p* < 0.001), and F (*p* = 0.016) were associated with human *E. coli*. Phylogroup B1 was also significantly associated with *E. coli* from soil (*p* < 0.001) and prawns (*p* < 0.001). On the contrary, phylogroup B2 was not specifically associated with *E. coli* with any source (*p* > 0.05). Similarly, we did not find any substantial association of any phylogroup with water (*p* > 0.05).

The varied distribution of the different phylogroups called for a need for diversity analysis. For this, the Shannon and Simpson diversity indices were calculated. As shown in Table 5, the highest diversity indices were observed for human isolates (Shannon index = 1.27, Simpson index = 0.64) and for animals (Shannon index = 1.20, Simpson index = 0.63) while the lowest diversity was found for soil (Shannon index = 0.27, Simpson index = 0.15). The Shannon Diversity indices of *E. coli* from humans was 1.27, and for non-human sources, it was 1.16—the difference in diversity being statistically significant (*p* > 0.05). Likewise, in the case of human (1.27) and animal (1.20) isolates, the difference in diversity was found to be statistically significant (*p* > 0.05).

### 3.4. Correspondence Analysis

**Figure 2:**
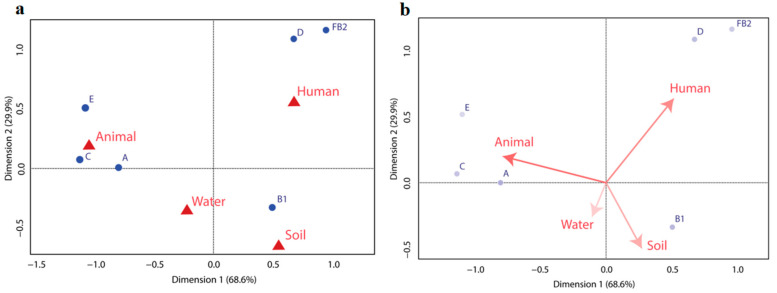
Correspondence analysis. (**a**) The bi-dimensional graph represents 98.43% of the total variation, with 68.58% explained by first dimension, and 29.85% by the second dimension. The phylogenetic groups are represented as blue/purple circle, whereas the isolate habitats are represented as red triangles/arrows. (**b**) In case of the asymmetric map, the length and brightness intensity of the circles correlates with the occurrence of the phylogenetic groups. The arrows between any two samples and the origin (the point of intersection of the dimensions) created angles. The sharper the angle, the more related the habitats are in case of *E. coli* phylogenetic group distribution.

In the symmetric map (Figure 2a), we can see that the B2, D, and F phylogroups almost extensively belong to humans, while A, C, and E phylogroups are predominant in animals. Phylogroup B1 was the most prevalent in soil, water, and prawn. The predominant group was B1, followed by A and D. The least predominant phylogroups were B2 and F, which overlapped with the B2 circle and E in the asymmetric map. Humans and animals showed more relatedness compared to humans and other sources of isolates. On the other hand, *E. coli* isolated from soil, water, and prawn showed similarity in the case of phylogroup distribution (Figure 2a,b).

## 4. Discussion

Different factors, such as climate, host body mass and diet, bacterial characteristics in different regions under antibiotic usage, and/or host genetic factors, influence the ecological structure and the distribution of *E. coli* phylogroups [27,28]. In our study, we observed a predominance of phylogroup B1 among the test isolates followed by phylogroup A. Furthermore, ref. [14] it has been previously confirmed that the bulk of the *E. coli* strains that can persist in the environment fit to the B1 phylogenetic group. Several researchers have also suggested that phylogroup A is best adapted to different environments [29,30]. In this study, *E. coli* isolated from animals mostly belonged to group A, while isolates from humans, prawns, water, and soil were mainly of phylogroup B1. Among non-human (animal, prawn, soil, and water) *E. coli* isolates, phylogroup B1 covered 45.32%, whereas, in human host isolates, it was 53.0%. Among non-human isolates, 35.25% belonged to phylogroup A as opposed to only 6.67% in human *E. coli*. This finding was particularly noticeable as phylogroup B1 represents persistent and naturalized environmental strains of *E. coli* [13,14]. However, in tropical areas, both groups A and B1 are widespread among human strains [31]. In one research [16], B1 was established as the main phylogroup of *E. coli* isolated from domestic animals, followed by phylogroup A. In contrast to these studies, we found phylogroup A (56.0%) as the dominating group in animal. Our study grouped 53.0% of human isolates into phylogroup B1. Phylogroups A and B1 have also been identified as causes of diarrheal diseases in humans [12]. In our research, 40% of the isolates from tap and surface water were grouped under phylogroup A. Similar results were reported earlier [32]; 44% of 150 isolates from raw wastewater were grouped under phylogroup A. Phylogenetic group E was significantly associated with animal sources.

The isolated *E. coli* strains were highly resistant to tetracyclines, ciprofloxacin, azithromycin, and amoxicillin-clavulanic acid. Among the B1 isolates (48.22%), only 29.5% were found to be MDR *E. coli*. Phylogroup A (26.9%) encompassed only 22.6% MDR *E. coli*. Only 2.03%, 9.14%, and 2.03% of total *E. coli* were characterized under phylogroups B2, D, and F, respectively. Although one B2 isolate was MDR, 100% F and 61.1% D isolates were found to be MDR. These are significant findings because extra-intestinal and virulent *E. coli* are classified under B2 and D phylogenetic groups and carriage of MDR can worsen infections by pathogenic strains. Moreover, phylogenetic group F is characterized as a sister group of B2 and is thus considered pathogenic. Interestingly, all MDR *E. coli* of phylogroup B2, D, and F were isolated from human sources. In the case of human isolates (n = 60), phylogroups B2 and D comprised 5% (n = 3) and 26.67% (n = 16), respectively. A similar distribution was reported by Stoppe et al., who reported 16.4% (n = 19) and 30.2% (n = 35) phylogroups B2 and D among 116 human host isolates [32]. In concordance with our study, a similar prevalence pattern was reported in a previous study [33], which revealed that phylogroup B1 (64%) and A (22%) were the most prevalent groups, followed by group D (11%) and group B2 (4%). Several previous studies also reported that phylogroup B1 was the predominant phylogroup isolated from feces of domestic and wild animals as well as from soil and surface water samples [18,34,35,36,37]. Phylogroup B1 dominance is the consequence of its extended survival in the environment that can be explained either by a unique set of stress tolerance traits [34,37,38] or the existence of some clades in B1 appearing readily in sediment and/or soil habitats [14].

However, diversity indexes (Shannon and Simpson Diversity indexes) showed that *E. coli* strains isolated from humans had greater diversity than in non-human hosts. In our study, the highest diversity was found in humans as was reported earlier [15]. Moreover, there was greater diversity in *E. coli* strains isolated from animals than in other non-human isolates. Humans and animals share some characteristics, such as diet and gut morphology, which may account for the differences in the diversity indexes.

Our results indicate that phylogenetic groups A, C, and E are more prevalent in *E. coli* isolated from the animal. Both the chi-square test and correspondence analysis showed a significant association of these three phylogroups with *E. coli* isolated from animals. Moreover, the prevalence of phylogroup B1 is also high in *E. coli* isolated from prawns, soil, and water. Both chi-square and CA agreed and showed a significant association of B1 with *E. coli* isolated from prawn and soil sources. In addition, neither test showed any clear association of B1 with *E. coli* isolated from water sources. For human sources, both chi-square and CA showed a significant association of phylogroup D and F with isolated *E. coli*. Although phylogroup B1 is found to be associated with human *E. coli* strains based on the chi-square test, CA showed no clear association. Both chi-square and the CA results showed a significant association of phylogenetic group E with animal sources. This finding is supported by the fact that phylogroup E is associated with *E. coli* O157:H7. *E. coli* O157:H7 is reported to be found in the intestinal content of some cattle, goats, and sheep [39,40].

Different phylogenetic groups of *E. coli* have been found in specific hosts and demonstrated the same level of adaptability to environmental conditions. In our study, it was important to determine if the isolates reached a particular environment by recent fecal contamination because a bacteria of fecal origin might be either commensal or pathogenic instead of environmental. Based on the absence of the *eae* gene in any of the *E. coli* isolates, our study suggests that these phylogenetic groups of *E. coli* were adapted to survive in their particular environments/sources.

## 5. Conclusions

In conclusion, this study investigated the prevalence rate, antibiotic resistance pattern, phylogenetic groups, and association with the hosts/sources of *E. coli* isolated from different environmental and clinical samples. We observed a total prevalence of 51.4% *E. coli* from various sources. The highest prevalence of *E. coli* was found for human clinical samples, and the lowest was for water samples. Neither of the isolates contained the *eae* gene, an indicator of recent fecal contamination, which confirms the presence of the isolates in the environment over a reasonably long time and indicates adaptability. Among these *E. coli* strains, phylogenetic group B1 was the principal phylogroup in different environments, with 29.5% of the isolates being MDR. Phylogroup B1 was significantly associated with human, prawn, and soil samples. Virulent phylogroups of *E. coli* (D and F) were mainly from the dynamic movement and adaptability of environmental *E. coli* (phylogroup B1) in different environments, including the human gut. This may establish that the demarcation line between environmental and commensal *E. coli* is questionable.

## Figures and Tables

**Table 1:** Source Distribution of samples collected in this study.

Source	Collection Sites
Human (n = 60)	Clinical samples from Medinova Diagnostic Centre, Dhaka, Bangladesh
Animal (n = 80)	Fecal specimen of animals
Prawn (n = 65)	Collected from local markets of Dhaka city
Soil (n = 85)	Different locations in Dhaka, Bangladesh
Water (n = 93)	Surface (n = 33) and tap water (n = 60), Dhaka, Bangladesh

**Table 2:** Antibiotic resistance phenotype of *E. coli* isolated from different samples.

Antibiotic Class	Antibiotic Agent	n (%) ^a^					
		Human (n = 60)	Animal (n = 54)	Prawn (n = 32)	Soil (n = 26)	Water (n = 2)	Total (n = 197)
Nitroheterocyclincs	Nitrofurantoin	2 (3.3)	15 (27.7)	8 (25.0)	0 (0)	1 (4.0)	26 (13.2)
Phenicols	Chloramphenicol	2 (3.3)	23 (42.5)	0 (0)	0 (0)	3 (12.0)	28 (14.2)
Tetracyclines	Tetracycline	22 (36.6)	35 (64.8)	7 (21.8)	4 (15.3)	3 (12.0)	71 (36.0)
B-lactams	Amoxicillin-Clavulanic acid	13 (21.6)	31 (57.4)	4 (12.5)	1 (3.8)	4 (16.0)	53 (26.9)
	Ceftriaxone	23 (38.3)	2 (3.7)	2 (6.2)	3 (11.5)	7 (28.0)	37 (18.7)
Quinolones	Ciprofloxacin	34 (56.6)	21 (38.8)	5 (15.6)	1 (3.8)	5 (20.0)	66 (33.5)
Aminoglycosides	Gentamicin	21 (35.0)	1 (1.8)	5 (15.6)	2 (7.6)	6 (24.0)	35 (17.7)
Macrolides	Azithromycin	36 (60.0)	10 (18.5)	8 (25.0)	4 (15.3)	6 (24.0)	64 (32.4)

^a^ Each column represents the number and percentages of isolates from each type of sample out of the total number of *E. coli* isolated (n).

**Table 3:** Association of Multidrug-resistant *E. coli* with different sample sources.

Sample Source	Multi-Drug Resistance		Association	
	Resistant to ≥Three Classes of Drugs	Resistant to <Three Classes of Drugs	χ^2^	*p*-Value
Human	31 (51.67)	29 (48.33)	29.853	0.001
Animal	28 (51.85)	26 (48.15)		
Prawn	5 (15.63)	27 (84.38)		
Soil	2 (7.69)	24 (92.31)		
Water	5 (20.0)	20 (80.0)		
Total	71 (36.0)	126 (64.0)		

**Table 4:** Prevalence of phylogenetic groups in *E. coli* and multidrug-resistant *E. coli* in respective phylogenetic group.

Phylogenetic Groups	Frequency n (%) ^a^	Multidrug-Resistant *E. coli* n (%) ^b^
A	53 (26.9)	12 (22.6)
B1	95 (48.22)	28 (29.5)
B2	4 (2.03)	1 (25.0)
C	17 (8.63)	11 (64.7)
D	18 (9.14)	11 (61.1)
E	6 (3.05)	4 (66.7)
F	4 (2.03)	4 (100.0)

^a^ Percentage of phylogenetic group out of total *E. coli* isolates (N = 197), ^b^ Percentage of MDR *E. coli* isolates out of respective phylogenetic groups (row).

**Table 5:** Distribution of phylogenetic groups among *E. coli* isolated from different samples.

Samples	Phylogenetic Groups, N (%)							Diversity Indexes	
	A	B1	B2	C	D	E	F	Shannon	Simpson
Human (n = 60)	4 (7)	32 (53) *	3 (5)	0	16 (27) *	1 (2)	4 (7) *	1.27	0.64
Animal (n = 54)	30 (56) *	4 (7)	0	13 (24) *	2 (4)	5 (9) *	0	1.2	0.63
Prawn (n = 32)	5 (16)	25 (78) *	0	2 (6)	0	0	0	0.66	0.37
Soil (n = 26)	4 (15)	21 (81) *	1 (4)	0	0	0	0	0.27	0.15
Water (n = 25)	10 (40)	13 (52)	0	2 (8)	0	0	0	0.91	0.59

* indicates significant association (*p* < 0.05) between phylogenetic groups of *E. coli* and different samples.

## Data Availability

Relevant data will be provided upon reasonable request to the corresponding author.

## Supplementary Materials

The following supporting information can be downloaded at: https://www.mdpi.com/article/10.3390/tropicalmed8040226/s1.
